# Time to Relapse Following Rituximab Therapy in Rheumatoid Arthritis and Its Determinants: A Single-Center Observational Study

**DOI:** 10.7759/cureus.110939

**Published:** 2026-06-15

**Authors:** Ikram El Moubarik, Imane El binoune, Samira Rostom, Ghizlane Ghoullam, Bouchra Amine, Salma Zemrani, Zhor Zeghari, Rachid Bahiri

**Affiliations:** 1 Rheumatology A, El Ayachi Hospital, Ibn Sina University Hospital (CHU Ibn Sina), Rabat, MAR; 2 Public Health, Laboratory of Community Health, Preventive Medicine, and Hygiene, Faculty of Medicine and Pharmacy, University Mohammed V, Rabat, MAR; 3 Laboratory of Biostatistics, Clinical Research, and Epidemiology, Faculty of Medicine and Pharmacy, University Mohammed V, Rabat, MAR; 4 Rheumatology, El Ayachi Hospital, Ibn Sina University Hospital (CHU Ibn Sina), Rabat, MAR

**Keywords:** b-cell depletion, biologic therapy, disease activity, retreatment strategy, rheumatoid arthritis, rituximab, time to relapse

## Abstract

Background: Rituximab (RTX), a monoclonal antibody targeting CD20, is effective in rheumatoid arthritis (RA) refractory to conventional and biological disease-modifying antirheumatic drugs (DMARDs). However, treatment response is not always sustained, and relapse may occur after variable intervals. Time to relapse (TTR) represents the duration during which RTX maintains clinical and biological efficacy before disease recurrence.

Objectives: This study aimed to evaluate TTR after the most recent RTX cycle in patients with RA and to identify the clinical, biological, and therapeutic factors associated with its duration.

Methods: We conducted a retro-prospective, single-center observational study including patients with RA treated with RTX between 2021 and 2025. The primary outcome was physician-assessed objective TTR following the most recent RTX cycle. Relapse-free survival was estimated using Kaplan-Meier analysis. Univariable and multivariable linear regression analyses were performed to identify clinical, biological, and treatment-related factors associated with TTR. Patient-reported relapse was collected as a secondary outcome and analyzed separately to allow comparison with physician-assessed relapse timing.

Results: A total of 97 patients were included, of whom 84 (84/97, 86.6%) were female. The mean age was 57 ± 10 years, and the median disease duration was 16 years (interquartile range (IQR) 10-22). RTX was administered as retreatment in 75 patients (75/97, 77.3%) and as first-line biologic therapy in 22 patients (22/97, 22.7%). The median objective TTR was nine months (IQR 7-11), which was similar to the patient-reported TTR of 9 months (IQR 6-11).

One-year flare-free survival was observed in 36 patients (36/97, 37.1%). In univariable analysis, lower baseline swollen joint count (SJC) and erythrocyte sedimentation rate (ESR) were associated with longer TTR. In multivariable analysis, only SJC remained independently associated with TTR (*P* = 0.040), whereas ESR showed a nonsignificant trend toward association.

Conclusions: The median TTR following RTX therapy in RA was approximately nine months. Baseline inflammatory burden, particularly SJC, emerged as the main factor associated with relapse timing, whereas RTX dose and number of treatment cycles were not significantly associated with TTR. These findings suggest that disease activity at the time of RTX administration may influence subsequent relapse timing. However, given the observational design and modest predictive performance of the final model, prospective studies are needed to confirm these observations and determine their implications for individualized retreatment strategies in RA.

## Introduction

Rheumatoid arthritis (RA) is a chronic systemic inflammatory disease that primarily affects synovial joints. It is characterized by joint pain, progressive structural damage, and functional disability, leading to impaired quality of life and a substantial socioeconomic burden [1].

RA is an autoimmune disorder involving complex interactions between multiple immune cell populations. Among these, B cells play a central role in disease pathogenesis through the production of pathogenic autoantibodies, antigen presentation to T cells, and secretion of pro-inflammatory cytokines [2,3].

Among B-cell-targeted therapies, rituximab (RTX) is the only biologic agent specifically approved for the treatment of RA. RTX is a chimeric anti-CD20 monoclonal antibody that induces selective depletion of CD20-positive B cells [4,5]. It was introduced into RA management in 2006, in combination with methotrexate (MTX), for patients with an inadequate response to at least one tumor necrosis factor (TNF) inhibitor [6]. Clinical trials have demonstrated its efficacy and safety, with a recommended dosing regimen consisting of two intravenous infusions of 1,000 mg administered on days 1 and 15 and repeated every 24 weeks [7].

According to European recommendations, RTX is indicated as a second-line biologic therapy. However, in Morocco, RTX is frequently used as a first-line biologic agent in patients with RA refractory to conventional synthetic disease-modifying antirheumatic drugs (csDMARDs), mainly because of its favorable safety profile in a tuberculosis-endemic setting.

Although fixed six-month retreatment intervals are generally considered optimal, clinical responses to RTX vary widely among patients. Several factors, including serological status, inflammatory biomarkers, and genetic background, may influence treatment durability. Peripheral B-cell reconstitution typically occurs between six and nine months after RTX administration [8,9], making the optimal retreatment timing challenging.

Several RTX retreatment strategies have been proposed, including regular retreatment at fixed intervals (every six months), retreatment at the time of clinical relapse, which corresponds to the strategy adopted in our local practice, and retreatment based on clinical deterioration within a treat-to-target approach [10]. However, considerable variability exists in the duration of response following RTX therapy, and reliable predictors of relapse timing remain insufficiently characterized. This knowledge gap complicates the optimization of retreatment schedules and underscores the need to identify factors associated with time to relapse (TTR) in routine clinical practice.

The TTR following RTX therapy corresponds to the period during which clinical efficacy gradually diminishes before the next infusion, mainly as a consequence of B-cell reconstitution and reactivation of autoimmune inflammation. Management of this phenomenon requires individualized therapeutic adjustments and close clinical and biological monitoring to maintain efficacy while minimizing the risks associated with prolonged immunosuppression.

This study aimed to evaluate the median TTR following the last RTX cycle in patients with RA and to identify the clinical, biological, and therapeutic factors associated with TTR duration.

## Materials and methods

Study design

We conducted a retro-prospective, observational, single-center study in the Rheumatology Department of Ibn Sina University Hospital, Rabat, Morocco, between January 2021 and March 2025. The study was conducted in accordance with ethical principles and approved by the local ethics committee (CERB 145-25). Written informed consent was obtained from all participants before inclusion.

Inclusion and exclusion criteria

Adult patients (≥18 years) diagnosed with RA according to the 2010 American College of Rheumatology/European League Against Rheumatism (ACR/EULAR) classification criteria and treated with RTX, regardless of the number of previous treatment cycles, were eligible for inclusion. For each patient, the most recent RTX cycle was considered for analysis.

Patients were excluded if they had severe cognitive impairment, declined participation, had incomplete follow-up data, were younger than 18 years, or experienced primary therapeutic failure, defined as a TTR of less than six months following the last RTX cycle. Eight patients met this criterion and were excluded from the analysis. This exclusion criterion was chosen to exclude primary non-responders and focus specifically on determinants of relapse among patients who achieved an initial clinical response to RTX.

Objectives and definition of relapse

The primary objective of the study was to assess the median TTR following the most recent RTX cycle. The secondary objective was to identify the clinical, biological, and therapeutic factors associated with TTR duration.

The primary study outcome, TTR, was defined as the time interval (in months) between the last RTX infusion and physician-assessed objective relapse. Objective relapse was determined based on recurrence of RA disease activity, taking into account disease activity scores (DAS28-CRP or DAS28-ESR), tender and swollen joint counts (SJCs), inflammatory biomarkers including erythrocyte sedimentation rate (ESR) and C-reactive protein (CRP), and the treating physician’s clinical judgment. No predefined DAS28 threshold, EULAR response criterion, or other standardized flare definition was prospectively applied. Relapse assessment reflected routine clinical practice and was based on the overall evaluation of clinical and biological disease activity.

Patient-reported subjective relapse was collected separately as a secondary measure for comparison with physician-assessed relapse. Subjective relapse was defined as patient-reported worsening of RA symptoms or perception of disease flare during routine clinical follow-up. Although visual analog scale (VAS) assessments were routinely recorded as part of standard clinical care, no predefined VAS threshold or validated flare-specific patient-reported outcome measure was used to define subjective relapse.

Data collection

Data were collected through patient interviews and electronic medical records. Retrieved variables included sociodemographic characteristics (age, sex, marital status, education level, occupation, and type of health coverage), RA-related characteristics (date of symptom onset, diagnosis date, disease duration, serological status including anti-cyclic citrullinated peptide antibodies (ACPAs) and rheumatoid factor (RF), erosive disease, and severe manifestations such as coxitis or atlantoaxial instability), comorbidities (hypertension, diabetes mellitus, dyslipidemia, cardiovascular disease, osteoporosis, stroke, menopause, depression, fibromyalgia, and tobacco use), therapeutic history (prior and current use of csDMARDs, previous biologic DMARD exposure, and corticosteroid use including dose and duration), and RTX-specific characteristics including number of previous RTX cycles, dosage and administration dates, date of the most recent cycle, date of disease flare, and calculated TTR in months.

RTX treatment protocol

RTX was administered according to three predefined treatment regimens. Patients initiating RTX therapy received the standard RA protocol consisting of two intravenous infusions of 1,000 mg administered on days 1 and 15. Patients who had previously received two or more treatment cycles generally received a reduced-dose regimen consisting of 500 mg administered on days 1 and 15. A small subset of patients received a low-dose protocol consisting of a single 500 mg infusion.

Disease activity and adverse events were systematically assessed at month 1 (M1), month 4 (M4), month 6 (M6), and monthly thereafter during follow-up. When treatment efficacy was maintained, a subsequent RTX infusion could be administered after a minimum interval of six months in the event of clinical worsening.

Statistical analysis

Statistical analyses were performed using Jamovi software version 2.3.28 (The Jamovi Project, Sydney, Australia). Continuous variables were expressed as mean ± standard deviation (SD) or median with interquartile range (IQR), depending on data distribution, whereas categorical variables were reported as numbers and percentages (*n*, %).

Categorical variables were compared using the chi-square test or Fisher’s exact test, as appropriate. Continuous variables were analyzed using Student’s t-test or Mann-Whitney U test according to data distribution. Kaplan-Meier survival curves were generated to assess relapse-free survival over time following RTX therapy.

Given that the primary objective was to identify factors associated with variations in the observed duration of response following RTX therapy, TTR was analyzed as a continuous variable. Univariable and multivariable linear regression analyses were subsequently performed to identify factors associated with prolonged TTR. Variables with *P* < 0.20 in univariable analysis were entered into the multivariable model. Patients with incomplete follow-up data were excluded during the selection process. Consequently, no imputation of missing values was performed in the statistical analyses. A two-sided *P*-value < 0.05 was considered statistically significant.

## Results

Baseline characteristics

A total of 97 patients with RA were included in the study, of whom 84 were female (84/97, 86.6%) and 13 were male (13/97, 13.4%). The mean age was 57.7 ± 10.1 years, and the median body mass index (BMI) was 25.5 kg/m² (IQR 22.5-29.0).

Comorbidities were present in 81 patients (81/97, 83.5%), with osteoporosis being the most frequent condition, affecting 71 patients (71/97, 73.2%), followed by hypertension in 25 patients (25/97, 25.8%) and diabetes mellitus in 19 patients (19/97, 19.6%). Cardiac disease was reported in two patients (2/97, 2.1%). Severe RA manifestations included coxitis in 10 patients (10/97, 10.3%) and atlantoaxial involvement in two patients (2/97, 2.1%).

The median disease duration was 16 years (IQR 10-22), and seropositivity for rheumatoid factor (RF) and/or anti-cyclic citrullinated peptide antibodies (anti-CCP) was identified in 94 patients (94/97, 96.9%).

Regarding csDMARDs, 36 patients (36/97, 37.1%) were not receiving any csDMARD at the time of assessment. MTX was used in 28 patients (28/97, 28.9%), sulfasalazine in 23 patients (23/97, 23.7%), and leflunomide in 10 patients (10/97, 10.3%).

Corticosteroid therapy was prescribed in 75 patients (75/97, 77.3%), with a median prednisone-equivalent dose of 5 mg/day (IQR 3-10). Previous exposure to biologic DMARDs was reported in seven patients (7/97, 7.2%) (Table 1).

**Table 1:** Baseline characteristics of the study population (n = 97). Data are presented as mean ± standard deviation (SD), median (interquartile range (IQR)), or number (%), as appropriate. BMI, body mass index; RF, rheumatoid factor; anti-CCP, anti-cyclic citrullinated peptide; TJC, tender joint count; SJC, swollen joint count; ESR, erythrocyte sedimentation rate; CRP, C-reactive protein; csDMARDs, conventional synthetic disease-modifying antirheumatic drugs; MTX, methotrexate; LFL, leflunomide; SLZ, sulfasalazine

Variable	Values
Age (years)	57.7 ± 10.1
BMI (kg/m²)	25.5 [22.5-29.0]
Sex	
Female, *n* (%)	84 (86.6)
Male, *n* (%)	13 (13.4)
Comorbidities, *n* (%)	81 (83.5)
Hypertension	25 (25.8)
Diabetes mellitus	19 (19.6)
Heart disease	2 (2.1)
Osteoporosis	71 (73.2)
Coxitis	10 (10.3)
Atlanto-axial involvement	2 (2.1)
Disease duration (years)	16 (10-22)
Seropositivity (RF and/or anti-CCP), *n* (%)	94 (96.9)
TJC	6 (3-10)
SJC	5 (3.7-7.0)
ESR (mm/hour)	33 (20-55)
CRP (mg/L)	14.6 (7.1-26.3)
Current csDMARDs, *n* (%)	
• None	36 (37.1)
• MTX	28 (28.9)
• LFL	10 (10.3)
• SLZ	23 (23.7)
Corticosteroid therapy, *n* (%)	75 (77.3)
Prednisone dose (mg/day)	5 [3-10]
Previous biologic DMARDs, *n* (%)	7 (7.2)

RTX treatment characteristics

RTX was administered as retreatment in 75 patients (75/97, 77.3%) and as first-line biologic therapy in 22 patients (22/97, 22.7%). The most frequently used dosing regimen was 500 mg × 2, administered in 66 patients (66/97, 68.0%), followed by 1 g × 2 in 29 patients (29/97, 29.9%). A minority of patients (2/97, 2.1%) received a single 500 mg infusion as part of an internal low-dose protocol.

The number of RTX cycles ranged from one to eight. During follow-up, disease flare occurred in 65 patients (65/97, 67.0%) (Table 2).

**Table 2:** Number of rituximab cycles and dosage regimens. Data are presented as median (IQR) or number (%), as appropriate. RTX, rituximab; TTR, time to relapse; IQR, interquartile range

Variable	*n* (%)
Number of RTX cycles	
1 cycle	22 (22.7)
2 cycles	21 (21.6)
3 cycles	24 (24.7)
4 cycles	12 (12.4)
≥5 cycles	18 (18.5)
Dosage regimens	
500 mg × 1	2 (2.1)
500 mg × 2	66 (68.0)
1 g × 2	29 (29.9)
Median objective TTR (months)	9.00 (7.75–11.3)
Median subjective TTR (months)	9.00 (6–11)
Flare events, *n* (%)	65 (67.0)

Time to relapse

The median physician-assessed (objective) TTR was 9 months (IQR 7-11), whereas the median patient-reported (subjective) TTR was also 9 months (IQR 6-11). No statistically significant difference was observed between the two assessments (*P* = 0.06).

TTR tended to be slightly longer among patients receiving retreatment compared with those initiating RTX therapy (median 10 months [IQR 6-30] versus 9 months (IQR 6-11), respectively), although this difference did not reach statistical significance (*P* = 0.202) (Table 3).

**Table 3:** Mean TTR according to the number of rituximab cycles. Data are presented as mean ± SD. RTX, rituximab; SD, standard deviation; TTR, time to relapse

Number of RTX cycles	Mean TTR ± SD (months)	*P*-value
1 cycle	8.73 ± 1.91	
2 cycles	10.7 ± 4.59	
3 cycles	12.5 ± 6.66	
4 cycles	9.25 ± 1.91	
≥5 cycles	10.8 ± 3.37	
Kruskal–Wallis test		p = 0.323

Kaplan-Meier analysis demonstrated that 36 patients (36/97, 37.1%) remained relapse-free one year after the most recent RTX cycle, corresponding to an estimated one-year flare-free survival rate of 37.1%.

Although TTR appeared to increase slightly with the number of RTX cycles, ranging from 8.73 ± 1.91 months after the first cycle to 12.5 ± 6.66 months after the third cycle, this variation was not statistically significant (Kruskal-Wallis test, *P* = 0.323) (Table 3).

Factors associated with TTR

Univariable linear regression analysis demonstrated that lower baseline SJC and lower ESR at the time of RTX infusion were associated with longer TTR (SJC: *β* = -0.317, 95% confidence interval (CI) -0.585 to -0.05, *P* = 0.021; ESR: *β* = -0.04, 95% CI -0.07 to -0.003, P = 0.034). No statistically significant associations were identified for age, sex, BMI, disease duration, seropositivity, corticosteroid use, csDMARD exposure, previous biologic therapy, or RTX dosing regimen (Table 4).

**Table 4:** Univariable linear regression analysis of factors associated with TTR. Results are presented as *β* coefficients with 95% confidence intervals (CIs). TTR, time to relapse; BMI, body mass index; TJC, tender joint count; SJC, swollen joint count; ESR, erythrocyte sedimentation rate; CRP, C-reactive protein; csDMARDs, conventional synthetic disease-modifying antirheumatic drugs; MTX, methotrexate; LFL, leflunomide; SLZ, sulfasalazine; RTX, rituximab; TNF, tumor necrosis factor; IL-6, interleukin 6

Variable	*β* coefficient	95% CI	*P*-value
Age	-0.03	(-0.13 to 0.07)	0.567
Sex (male vs. female)	-0.78	(-4.08 to 2.51)	0.637
BMI	0.01	(-0.21 to 0.22)	0.960
Disease duration	-0.02	(-0.13 to 0.09)	0.655
Seropositivity	-0.82	(-9.65 to 8.01)	0.853
Corticosteroid therapy (yes/no)	0.33	(-2.23 to 2.89)	0.797
Prednisone dose (mg/day)	-0.19	(-0.54 to 0.15)	0.279
Previous biologic therapy	-2.59	(-7.07 to 1.87)	0.251
TJC	-0.157	(-0.34 to 0.03)	0.100
SJC	-0.317	(-0.585 to -0.05)	0.021
ESR (mm/h)	-0.04	(-0.07 to -0.003)	0.034
CRP (mg/L)	-0.02	(-0.05 to 0.01)	0.304
csDMARDs			
MTX vs. None	0.639	(-1.89 to 3.17)	0.616
LFL vs. None	3.282	(-0.63 to 7.19)	0.099
SLZ vs. None	-0.818	(-3.62 to 1.98)	0.562
Previous biologic type			
Anti-TNF vs. Anti-IL6	1	(-9.65 to 11.7)	0.852
None vs. anti-IL6	4.35	(-4.41 to 13.1)	0.325
RTX dose			
500 mg × 1 vs. 1 g × 2	0.553	(-5.90 to 7.01)	0.865
500 mg × 2 vs. 1 g × 2	1.776	(-0.585 to 4.14)	0.138
Number of RTX cycles			
2 vs. 1	2.0	(-1.07 to 5.08)	0.199
≥3 vs. 1	0.57	(-2.22 to 3.37)	0.685

In multivariable linear regression analysis adjusted for potential confounders, higher baseline SJC remained independently associated with shorter TTR (adjusted *β* = -0.30, 95% CI -0.58 to -0.015, *P* = 0.040), whereas elevated ESR demonstrated a non-significant trend toward earlier relapse (adjusted *β* = -0.034, 95% CI -0.073 to 0.005, *P* = 0.089). Neither the number of previous RTX cycles nor the dosing regimen was independently associated with TTR. The final model explained a modest proportion of TTR variability (adjusted *R*² = 0.159) (Table 5).

**Table 5:** Multivariable linear regression analysis of predictors of objective TTR. Results are presented as *β* coefficients with 95% confidence intervals (CIs). TTR, time to relapse; SJC, swollen joint count; ESR, erythrocyte sedimentation rate; RTX, rituximab; CI, confidence interval

Variable	Estimation *β*	Standard error	95% CI	t	*P*-value
Intercept	12.96	1.65	(9.66-16.25)	7.86	<0.001
SJC	-0.30	0.14	(-0.58 to -0.015)	-2.10	0.040
ESR	-0.034	0.020	(-0.073 to 0.005)	-1.73	0.089
Previous biologic therapy (yes vs. no)	-2.49	2.22	(-6.93 to 1.96)	-1.12	0.268
Rituximab dose 500 mg ×1 vs. 1 g ×2	2.27	3.26	(-4.24 to 8.79)	0.70	0.488
Rituximab dose 500 mg ×2 vs. 1 g ×2	0.61	1.21	(-1.79 to 3.01)	0.51	0.616

## Discussion

In this retro-prospective observational study, we evaluated the TTR following the most recent RTX cycle in patients with long-standing RA and explored factors associated with its duration. We observed a median TTR of nine months, with marked interindividual variability, and identified baseline inflammatory activity, particularly SJC, as the principal determinant of relapse timing.

TTR after RTX

The median TTR observed in our cohort is consistent with real-world studies reporting retreatment or relapse intervals ranging from six to twelve months when RTX is administered using an on-demand strategy rather than fixed retreatment intervals [4,11]. While pivotal randomized controlled trials primarily demonstrated sustained efficacy up to 24 weeks, subsequent observational cohorts have shown considerable heterogeneity in RTX durability, with some patients maintaining disease control for more than one year whereas others experience earlier relapse [12].

In the large GERINIS observational cohort, retreatment intervals progressively shortened with successive RTX cycles, decreasing from a mean of 9.6 months between the first and second cycles to 7.7 months between the fifth and sixth cycles, highlighting the variability in RTX durability observed in routine clinical practice [13].In our study, 36 patients (36/97, 37.1%) remained flare-free one year after the last RTX cycle, a proportion comparable to that reported in real-world cohorts managed with relapse-driven retreatment strategies [4,13].

We also observed a nonsignificant trend toward longer TTR among patients receiving RTX as retreatment compared with those initiating therapy. Similar findings have been reported in observational studies, suggesting that repeated RTX exposure does not necessarily result in progressive shortening of response duration [14].

Baseline inflammatory activity as the main determinant of relapse

The most robust finding of our study was the independent association between baseline SJC and TTR. Patients with higher SJC at the time of RTX infusion experienced earlier relapse, even after adjustment for potential confounders. This observation is consistent with previous studies demonstrating that higher baseline disease activity and incomplete suppression of synovitis are associated with reduced durability of RTX response [15,16].

Baseline ESR was associated with TTR in univariable analysis but did not remain statistically significant in the multivariable model, although a consistent trend persisted. This finding suggests that clinical measures of inflammation, particularly SJC, may better reflect the residual inflammatory burden driving relapse after B-cell depletion than isolated laboratory markers. Similar conclusions have been reported in studies emphasizing the importance of achieving remission or low disease activity at the time of retreatment [15].

From a pathophysiological perspective, relapse after RTX is generally attributed to peripheral and tissue B-cell reconstitution. However, several studies have demonstrated that clinical relapse does not systematically coincide with B-cell repopulation, suggesting that residual synovial inflammation and qualitative alterations in B-cell subsets may substantially contribute to relapse timing [12]. These findings support the concept that both quantitative and qualitative aspects of B-cell biology influence treatment durability.

Although multiple demographic, clinical, laboratory, and treatment-related variables were evaluated, the final model explained only a modest proportion of the variability in TTR (adjusted *R*² = 0.159). This suggests that additional determinants of relapse timing were not captured in the present study. Potential contributors include peripheral B-cell repopulation kinetics, immunoglobulin levels, genetic factors, and other immunological characteristics that were not routinely available in our cohort. Future studies incorporating biological and immunological monitoring may help improve prediction of RTX durability and relapse timing.

Serological status and treatment-related outcomes

Contrary to studies reporting more sustained responses among RF- or ACPA-positive patients [12,16], serological status was not associated with TTR in our cohort. This discrepancy may be explained by the very high prevalence of seropositivity in our study population, thereby limiting discrimination between serological subgroups.

Similarly, no association was observed between TTR and RTX dose or the number of previous RTX cycles. These findings are consistent with studies suggesting comparable clinical durability between standard-dose (1 g × 2) and reduced-dose (500 mg × 2) RTX regimens in selected patients [17]. This observation may have important implications for optimizing RTX use, particularly in settings where treatment cost and long-term safety remain important considerations.

Although no statistically significant differences were observed according to retreatment status or number of RTX cycles, the observed differences in median and mean TTR were small and of uncertain clinical relevance. In addition, the limited sample size within several treatment subgroups may have reduced the precision of effect estimates and limited the ability to detect modest differences between groups.

Concomitant csDMARD use and corticosteroid therapy were likewise not associated with prolonged TTR, supporting the hypothesis that RTX durability in long-standing RA may be influenced more strongly by disease-related factors than treatment-related parameters. Differences compared with studies reporting a beneficial effect of concomitant csDMARDs may be explained by variations in study populations, disease chronicity, and retreatment strategies across cohorts [14].

Objective versus patient-reported relapse

An original aspect of this study was the comparison between objective and patient-reported TTR. The absence of a statistically significant difference between physician-assessed and patient-reported TTR indicates that no significant difference was detected between the two measures at the group level. However, no formal agreement analysis was performed; therefore, concordance between physician-assessed and patient-reported relapse cannot be established. Nevertheless, patient perception of disease activity may provide complementary information alongside physician assessment and could potentially contribute to individualized RTX retreatment strategies, as advocated in treat-to-target approaches [18]. Further studies using standardized and validated patient-reported outcome measures are warranted to confirm these observations.

Clinical implications

Our findings suggest that an individualized, disease activity-guided RTX retreatment strategy may be preferable to systematic fixed-interval retreatment. Patients with a higher inflammatory burden, particularly elevated SJC, may warrant closer follow-up and earlier reassessment following RTX infusion. Conversely, patients achieving lower inflammatory activity may benefit from extended retreatment intervals, potentially reducing cumulative immunosuppressive exposure. However, prospective studies are needed to confirm these observations and to determine their implications for retreatment decision-making.

In tuberculosis-endemic settings, where RTX is frequently preferred because of its favorable infectious safety profile, optimization of retreatment timing based on disease activity may further improve the benefit-risk balance of therapy [11,19].

Limitations

This study has several limitations. First, its single-center design and moderate sample size may limit the generalizability of the findings. Second, peripheral B-cell counts and immunoglobulin levels were not systematically available, precluding evaluation of their predictive value for TTR despite evidence supporting their potential relevance [20]. Third, patients experiencing relapse within six months of RTX administration were excluded because they were considered primary treatment failures (*n* = 8). While this approach allowed us to focus on secondary loss of response, it may have introduced selection bias, slightly overestimated the observed median TTR, and limited the applicability of our findings to the broader population of patients treated with RTX. Fourth, although patient-reported relapse was collected for comparison with physician-assessed relapse, no validated patient-reported outcome measure or standardized flare assessment tool was used. However, subjective relapse was not used to define the primary study outcome, which was based on physician-assessed objective relapse. Fifth, although TTR was analyzed as a continuous outcome using linear regression to identify factors associated with variations in response duration, alternative survival-based multivariable approaches such as Cox proportional hazards regression could provide complementary information and account more directly for censoring. Finally, the observational nature of the study precludes causal inference, and residual confounding related to therapeutic decision-making cannot be excluded.

## Conclusions

In this real-world cohort of patients with long-standing RA, the median TTR following RTX therapy was nine months, with substantial interindividual variability. Baseline inflammatory burden, particularly SJC, emerged as the strongest predictor of TTR, emphasizing the potential role of residual synovial inflammation in disease recurrence. In contrast, neither RTX dose nor the number of previous treatment cycles was significantly associated with relapse timing, while concomitant csDMARD and corticosteroid use showed limited impact, suggesting that disease-related factors may contribute more substantially than treatment-related parameters to the durability of response.

These findings suggest that disease activity at the time of RTX administration may influence subsequent relapse timing and support the potential value of individualized, disease activity-guided follow-up strategies. SJC may represent a simple and pragmatic clinical marker for identifying patients at increased risk of earlier relapse. However, given the observational design of the study and the modest predictive performance of the final model, these findings should be interpreted with caution and require confirmation in prospective studies before being incorporated into retreatment decision-making.

Future prospective studies incorporating serial B-cell monitoring, immunoglobulin profiling, and comprehensive disease activity assessment are warranted to improve prediction of relapse timing and further refine individualized RTX retreatment strategies, particularly in patients with long-standing RA.
